# Access to routine primary healthcare and past-year dental visits: results from the 2017–2020 National Health and Nutrition Examination Survey

**DOI:** 10.1017/S146342362610125X

**Published:** 2026-05-27

**Authors:** Seana Camp, McKing Izeiza Amedari, Adejare (Jay) Atanda

**Affiliations:** 1 University of Pittsburgh Graduate School of Public Health, USA; 2 John D. Bower School of Population Health, University of Mississippi Medical Cen, USA; 3 RAND Corporation, Arlington, USA

**Keywords:** dental care, dental visits, oral health disparities, primary healthcare

## Abstract

**Aim:**

To examine the relationship between access to routine primary healthcare (PHC) and receipt of dental visits.

**Background:**

PHC plays a vital role in promoting overall health and connecting patients to preventive services. Regular dental visits are crucial for preventing oral health issues and managing chronic diseases associated with oral health. However, disparities in healthcare access may influence the likelihood of individuals receiving necessary dental care.

**Methods:**

Data from 8, 727 participants ≥ 20 years in the 2017–2020 National Health and Nutritional Examination Survey were used for cross-sectional analysis. Logistic regressions explored the relationship between PHC and ‘past-year dental visits’, while controlling for sociodemographics.

**Findings:**

84% of the study population had access to routine PHC, and 63% had a past-year dental visit. Adults with PHC were significantly more likely to have had a past-year dental visit (OR = 2.24; 95% CI: 1.76–2.84). This was true for women, the oldest adult age group, and those with the highest educational levels. Black study participants were less likely to have had a past-year dental visit (OR = 0.55; 95% CI: 0.54, 0.83), so were the unmarried and those without health insurance.

**Conclusion:**

Access to routine PHC significantly increases the likelihood of a past-year dental visit, with gender, age, and educational levels having strongest effects. However, Black adults remain less likely to utilize dental services, even with access to routine PHC and health insurance. Strengthening the physician-dentist patient partnership through integrated delivery models of care that embed oral health services with primary care could be key.

## Background

Despite the US Surgeon General identifying oral disease as a priority health concern and urging all health professionals to address it, there has been minimal progress in restructuring the healthcare delivery system to adequately meet the nation’s oral health needs and eliminate the artificial divide between oral and systemic health (National Institute of Dental and Craniofacial Research, 2021). Other calls to action further highlight oral healthcare as a social justice issue and highlight the need for collaborative programming and health policy support including building upon existing health safety nets to link oral health services with primary care (U.S. Department of Health and Human Services, 2003; Treadwell HM, 2007; Laurie Smith *et al*., 2024).

Access to oral healthcare at the population level is hindered by macro-level factors such as population-wide policies and systems in place that prevent the seamless and integrated delivery of oral and general health services (Prasad *et al*., 2019; Patro *et al*., 2024). Historically, dental professionals, including dentists, dental hygienists, and dental assistants have been trained, reimbursed, and practiced separately from their medical counterparts. This long-standing division has reinforced the disconnect between dentistry and other healthcare fields (Atchison *et al*., 2018; Northridge *et al*., 2020). With the exclusion of dentistry at the establishment of medical schools, there is minimal participation in the development of health policies or the involvement of dental professionals in the care of adults at the primary healthcare (PHC) level (McCauley *et al*., 2003; Northridge *et al*., 2020). Separate medical and dental systems exacerbate disparities in healthcare access for underserved groups who already face limited access to oral health services. This division also leads to disorganized service delivery for healthcare workers, ultimately resulting in poor oral health outcomes and worsening existing disparities (Northridge *et al*., 2020).

To provide whole-person, integrated, patient-centred, and comprehensive care, it is important to connect oral health with PHC because oral health directly affects overall health and quality of life. Integration has generally centred on providing some oral health services in PHC because primary care clinicians are well positioned to reduce the burden of oral diseases. This can involve integrating oral health into routine care by providing counselling on oral hygiene and diet, promoting smoking cessation, offering fluoride supplementation or application, and screening for dental diseases (Stephens *et al*., 2018).

The 2021 National Institute of Dental and Craniofacial Research report, ‘*Oral Health in America: Advances and Challenges*’ cites the achievements of dental programmes situated in health centres (National Institute of Dental and Craniofacial Research, 2021). These programmes served 5.7 million patients through 13.7 million visits, with dental sealant applications in children aged 6–9 increasing from 42% to 56% in 2021 (Health Resources and Service Administration 2024).There is value in considering PHC settings essential for integrating oral health services. More Americans lack dental insurance than medical insurance (Mascarenhas *et al*., 2020), making them more likely to visit their PHC provider for health issues linked to or originating from poor oral health, such as respiratory diseases, cardiovascular diseases, and diabetes (Stephens *et al*., 2018). For example, in a cohort of diabetic participants who had dental visits through primary care, such visits were associated with an approximate 0.5% improvement in glycated haemoglobin levels (Horbach *et al*., 2021). Similarly, a longitudinal cohort study using NHANES data highlighted the benefits of professional dental treatment for overall health, reporting a reduced risk of mortality associated with preventive dental visits (Xu *et al*., 2023). The authors also noted that poor dental visits for dental treatments were linked with higher risks for cause-specific and all-cause mortality. Although the findings come from a self-reported data source, which may be associated with some subjectivity, they are based on a large, nationally representative sample size with robust control of confounders and sensitivity analyses. Furthermore, a study on the implications of medical and dental integration in the United States highlighted missed opportunities for dental care among adults with chronic conditions who had medical visits in the previous year but no dental visits (Wei *et al*., 2022). The study also estimated that integrating oral healthcare into primary care could benefit approximately 50% more adults, as medical visits provide a key opportunity for delivering oral health services and making dental referrals.

Access to insurance significantly influences dental visits and the utilization of dental services. The dental insurance market is currently experiencing notable growth and transformation. While most healthcare systems do not provide dental insurance, some Health Maintenance Organizations (a type of health insurance plan focused on preventive care and offering services within a network of providers) are beginning to include dentistry as part of their covered medical services. This shift is often facilitated through Medicare Advantage Plans, approximately 65% of which now offer basic dental insurance coverage (Mascarenhas *et al*., 2020; Pope, 2016). This trend is expected to continue, as integrating dental insurance into medical insurance plans lowers overall costs. These savings stem from reduced administrative expenses and increased reliance on allied dental health professionals, such as dental hygienists (Mascarenhas *et al*., 2020; Pope, 2016).

Despite its significance, integrating oral health into primary care remains an emerging practice within healthcare services (Phillips *et al*., 2016). This paper provides an evidence basis to improve the integration of oral healthcare into primary care, given the critical linkages between oral health and many systemic diseases, both in their manifestation and treatment. The aim was to examine the relationship between access to routine PHC and past-year dental visits. We hypothesized that access to routine PHC may be associated with past-year dental visits.

## Methods

### Epidemiologic design and study population

This is a cross-sectional study and a retrospective analysis of 2017–2020 National Health and Nutrition Examination (NHANES) Survey data representative of US non-institutionalized adults aged 20 years and older. NHANES uses a multistage, stratified, clustered, probability sampling design to identify a nationally representative sample of non-institutionalized civilians in the USA (Stierman *et al*., 2021). Participants completed a household interview, laboratory measurements, and physical examinations, and the details of the survey design have been published previously (Stierman *et al*., 2021). This is a secondary analysis of data publicly available from NHANES; hence IRB and ethics approvals were waived according to guidelines of the National Center for Health Statistics (NCHS) Ethics Review Board which manages consent to participate in NHANES, with NCHS ERB Protocol Approval Number #2018-01. Individuals participating in NHANES have specifically consented to the release of their personal information.

### Dependent variable

The dependent or outcome variable was past-year dental visits, measured as a binary (yes/no) variable. In NHANES, dental visits were recorded as a categorical variable, which included visits to any healthcare provider for dental care, such as orthodontists, oral surgeons, dental hygienists, and other dental specialists across 10 categories. This was recoded into a binary variable, as shown in Table 1.

**Table 1.** Descriptive statistics and bivariate results for covariates by past-year dental visits, NHANES 2017–2020

	Total	No past-year dental visit	Has past-year dental visit	Chi-square	*p*-Value
Study sample	*n* = 8,727	*n* = 4,149	*n* = 4,578	–	–
Study population (weighted)	*n* = 229,421,678	–	–	–	–
Routine place for healthcare (Access to primary care)	–	–	–	53.59	0.0000
No routine primary healthcare	16% (1,393)	64% (906)	36% (481)	–	–
Has routine primary healthcare	84% (7,352)	37% (3,243)	63% (4,097)	–	–
Gender	–	–	–	38.16	0.0000
Male	48% (4,221)	46% (2,190)	54% (2,024)	–	–
Female	52% (4,524)	37% (1,959)	63% (2,554)	–	–
Age (years)	–	–	–	13.67	0.0053
20–64	74% (6,446)	43% (3,082)	57% (3,347)	–	–
65–74	15% (1,275)	34% (608)	66% (666)	–	–
75+	12% (1,024)	35% (459)	65% (565)	–	–
Race/ethnicity	–	–	–	65.83	0.0000
White (non-Hispanic)	37% (3,204)	38% (1,400)	62% (1,804)	–	–
Black (non-Hispanic)	28% (2,447)	52% (1,343)	48% (1,098)	–	–
Asian (non-Hispanic)	13% (1,113)	39% (428)	61% (682)	–	–
Hispanic	23% (1,981)	49% (978)	51% (994)	–	–
Educational status	–	–	–	207.54	0.0000
Less than H/S	19% (1,692)	60% (1,053)	40% (633)	–	–
High School/GED	24% (2,115)	50% (1,141)	50% (972)	–	–
More than H/S	56% (4,938)	34% (1,955)	66% (2,973)	–	–
Marital status	–	–	–	31.59	0.0001
Married	58% (5,031)	38% (2,171)	62% (2,854)	–	–
Unmarried (separated, widowed, divorced)	23% (2,027)	45% (1,068)	55% (955)	–	–
Never married	19% (1,687)	50% (910)	50% (769)	–	–
Health insurance status	–	–	–	79.41	0.0000
Has health insurance	58% (5,031)	37% (3,175)	63% (4,156)	–	–
No health insurance	23% (2,027)	69% (974)	31% (422)	–	–

*Note*. *Statistically significant *P*-values for the Chi-square tests are set at *p* < 0.05. All provided data are weighted using NHANES sample weights provided for 2017–2020.

### Independent variable

The *key* independent or *predictor* variable was access to a routine place for healthcare, described here as access to routine PHC, assessed as a binary (yes/no) variable. Access to routine PHC in NHANES was a categorical variable in six categories in answer to the question: ‘Is there a place that you usually go when you are sick, or you need advice about your health’? This was recoded into an *indicator*, binary variable as shown in Table 1.

### Covariates

These consisted of sociodemographic factors such as sex, age, race/ethnicity, education level, marital status, and health insurance status), as shown in Table 1.

### Statistical analysis

Frequencies and percentages were calculated to present a descriptive analysis. Bivariate analyses for comparing past-year dental visits (dependent variable) in the sample were performed using weighted Chi-square tests for the key independent variable (access to routine PHC) and covariates. This informed variable selection for our unadjusted logistic regression model.

The relationship between access to primary care and past-year dental visits was assessed using unadjusted and adjusted (multivariable analysis) logistic regression models resulting in odds ratios (ORs) and 95% confidence intervals (CIs). The multivariable analysis was adjusted for *all* sociodemographic factors that were statistically significant in the unadjusted logistic regression model – sex, age, race/ethnicity, education level, marital status, and health insurance status.

All survey data were weighted using NHANES provided survey weights to adjust for the differential probabilities of selection and non-response, making results generalizable. No imputation was performed for missing data, with complete case analysis utilized in all analyses. Analyses were conducted using Stata/MP version 17.0 (StataCorp LP, College Station, TX).

## Findings

### Descriptive results

There were 8,727 adult participants in our 2017–2020 NHANES analytic sample representing 230 million American adults ≥ 20 years old (weighted study population). In total, 84% of US adults had access to routine PHC, with 63% having had a past-year dental visit. In the study population, 52% were female, 74% were aged 20–64, 37% identified as White, 56% had an education level higher than high school, 58% were married, and 58% had health insurance (Table 1).

### Bivariate analysis results

Chi-square tests were conducted to evaluate the relationships between access to routine PHC, past-year dental visits, and covariates. All variables showed statistically significant results at *p* < 0.05 (Table 1).

### Multivariable and stratified analysis results

The main results are presented for both unadjusted and adjusted analyses. In the unadjusted regression (Table 2 - Model 1), all variables were significantly associated with past-year dental visits. Participants with access to routine PHC had three times the odds of having a past-year dental visit compared to those without access (unadjusted OR = 3.01; 95% CI: 2.44–3.73; *p* < 0.001).

**Table 2.** Unadjusted and adjusted analysis: odds ratios (ORs) and 95% confidence intervals (CIs) for past-year dental visits, NHANES 2017–2020

	Unadjusted – model 1		Fully adjusted – model 2**	
Variables	OR^#^	95% CI^$^	OR^#^	95% CI^$^
Routine place for primary care				
No routine access	**Ref**	–	–	–
Has routine access	3.01*	2.44, 3.73	2.24*	1.76, 2.84
Sex				
Male	**Ref**	–	–	–
Female	1.43*	1.03, 1.36	1.30*	1.15, 1.46
Age (years)				
Age 20–64	**Ref**	–	–	–
Age 65–74	1.44*	1.13, 1.82	1.12	0.86, 1.44
≥75 years	1.37*	1.11, 1.69	1.21*	0.94, 1.56
Race and ethnicity				
White (non-Hispanic)	**Ref**	–	–	–
Black (non-Hispanic)	0.55*	0.44, 0.68	0.67*	0.54, 0.83
Asian (non-Hispanic)	0.95	0.77, 1.18	0.97	0.77, 1.22
Hispanic	0.64*	0.53, 0.76	1.02	0.83, 1.24
Educational level				
Less than H/S	**Ref**	–	–	–
High school/GED	1.53*	1.29, 1.80	1.43*	1.20, 1.69
More than H/S	2.91*	2.47, 3.42	2.48*	2.00, 3.06
Marital status				
Married	**Ref**	–	–	–
Unmarried (separated, widowed, divorced)	0.75*	0.65, 0.87	0.72*	0.62, 0.84
Never married	0.62*	0.51, 0.75	0.74*	0.61, 0.91
Health insurance status				
Has health insurance	**Ref**	–	–	–
No health insurance	0.27*	0.22, 0.33	0.44*	0.35, 0.55

*Note*. Ref stands for reference category.

*Statistically significant ORs are set at *P* < .05.

# ORs: Odds ratios.

$ CI: Confidence interval at the 95% level.

**Model 2 was adjusted for sociodemographic variables (sex, age, race/ethnicity, education level and marital status), and health insurance status.

All provided data are weighted using NHANES sample weights provided for 2017–2020.

In the adjusted analysis (Table 2 – Model 2), all variables remained significantly associated with past-year dental visits. Participants with access to routine PHC had 2.24 times the odds of having a past-year dental visit compared to those without access (adjusted OR = 2.24; 95% CI: 1.76–2.84; *p* < 0.001). This was also true for women (adjusted OR = 1.30; 95% CI: 1.15–1.46; *p* < 0.001), those ≥75 years (adjusted OR = 1.21; 95% CI: 0.94–01.56; *p* < 0.001), and those with high school/GED (adjusted OR = 1.43; 95% CI: 1.20–1.69; *p* < 0.001) and more than high school (adjusted OR = 2.48; 95% CI: 2.00–3.06; *p* < 0.001) education. Black participants were 33% less likely to have a past-year dental visit (adjusted OR = 0.67; 95% CI: 0.54–0.83; *p* < 0.001). Those without health insurance were 56% less likely to have a past-year dental visit (adjusted OR = 0.44; 95% CI: 0.35–0.55; *p* < 0.001).

In stratified analysis by health insurance status, the strength, direction, and significance for the predictor, access to routine PHC remained largely unchanged (Table 3 – Models 3 and 4). Among those with health insurance (Table 3 – Model 3), participants with access to routine PHC had 2.26 times the odds of having a past-year dental visit compared to those without access (adjusted OR = 2.26; 95% CI: 1.70–2.84; *p* < 0.001). Among those with *no* health insurance (Table 3 – Model 4), participants with access to routine PHC had 2.14 times the odds of having a past-year dental visit compared to those without access (adjusted OR = 2.14; 95% CI: 1.52–3.01; *p* < 0.001). Importantly, in this stratified analysis, among those with health insurance (Table 3 – Model 3), Black participants continued to be less likely (35% less likely) to have a past-year dental visit (adjusted OR = 0.65; 95% CI: 0.52–0.80; *p* < 0.001).

**Table 3.** Stratified analysis by health insurance: odds ratios (ORs) and 95% confidence intervals (CIs) for past-year dental visits, NHANES 2017–2020

	Have health insurance – model 3**		Have no health insurance – model 4**	
Variables	OR^#^	95% CI^$^	OR^#^	95% CI^$^
Routine place for primary care				
No routine access	**Ref**	–	–	–
Has routine access	2.26*	1.70, 2.84	2.14*	1.52, 3.01
Sex				
Male	**Ref**	–	–	–
Female	1.27*	1.11, 1.46	1.53*	1.15, 2.04
Age (years)				
Age 20–64	**Ref**	–	–	–
Age 65–74	1.13	0.86, 1.47	0.64	0.24, 1.69
≥75 years	1.21	0.93, 1.56	3.51	0.87, 14.12
Race and ethnicity				
White (non-Hispanic)	**Ref**	–	–	–
Black (non-Hispanic)	0.65*	0.52, 0.80	0.91	0.58, 1.43
Asian (non-Hispanic)	0.94	0.76, 1.16	1.34	0.60, 3.00
Hispanic	0.97	0.81, 1.17	1.29	0.87, 1.94
Educational level				
Less than H/S	**Ref**	–	–	–
High school/GED	1.51*	1.23, 1.85	1.13	0.71, 1.80
More than H/S	2.66*	2.20, 3.22	1.69*	1.01, 2.81
Marital status				
Married	**Ref**	–	–	–
Unmarried (separated, widowed, divorced)	0.69*	0.58, 0.82	1.10	0.74, 1.62
Never married	0.73*	0.58, 0.91	0.92	0.65, 1.29
Health insurance status				
Has health insurance	**Ref**	–	–	–
No health insurance	–	–	–	–

*Note*. Ref stands for reference category.

*Statistically significant ORs are set at *P* < .05.

# ORs: Odds ratios.

$ CI: Confidence interval at the 95% level.

**Models 3 and 4 are a stratified analysis by health insurance status and adjusted for sociodemographic variables (sex, age, race/ethnicity, education level and marital status).

All provided data are weighted using NHANES sample weights provided for 2017–2020.

## Discussion

The overarching objective of this study was to highlight the effect having access to routine PHC has on past-year dental visits among US adults aged ≥ 20 years though we were able to parse out other results. Our analysis revealed that 84% of US adults have a regular source of PHC, which aligns with broader healthcare trends where approximately 90% of insured adults report having a usual source of care (Newacheck *et al*., 2000; Dubay *et al*., 2001; Institute of Medicine (US) Committee on the Consequences of Uninsurance *et al*., 2002; Stevens *et al*., 2006; Pleis *et al*., 2007). This study also shows that over half of US adults with access to routine PHC had a past-year dental visit, compared to only one-third with no access to routine PHC. This significant result supports our hypothesis that access to routine PHC increases the likelihood of past-year dental visits. Given that more Americans have medical insurance (91%) compared to dental insurance (77%), integrating oral care into routine primary care could improve and expand access to dental care (Mascarenhas *et al*., 2020). This impact is particularly evident in children, where having a personal healthcare provider has been shown to increase the likelihood of dental visits and access to preventive dental care (Martin *et al*., 2009).

Our study also shows that even with health insurance, Black participants were still less likely to have a past-year dental visit compared to their White counterparts. Access-to-care challenges are complex and do not have simple or immediate solutions. While more Americans are experiencing improved oral health, these benefits are not equally distributed. Disparities persist, particularly among low-income individuals, minority groups, and institutionalized elderly populations who often lack adequate access to care (Guay *et al*., 2004). Our study findings highlight this disparity among Black individuals. However, in contrast to published work, the oldest adults in our study were more likely to have had a “past-year dental visit” if they had access to routine PHC. We suspect this may be because these individuals are non-institutionalized.

PHC providers play a critical role as entry points for accessing dental care, largely due to the higher prevalence of medical insurance compared to dental insurance (Cruz *et al*., 2004). Understandably, having health insurance is distinct from the integration of medical and dental services; nevertheless, it improves access to care and reduces cost-related delayed dental visits (Amedari *et al*., 2025; Atanda *et al*., 2025), and may be a necessary pathway for cooperation and collaboration for the delivery of integrated medical and dental services. Moreover, enhanced reimbursement rates in Medicaid may increase dentists’ participation (Cruz *et al*., 2004). However, the absence of a primary care provider is a significant risk factor for not receiving preventive dental care, therefore, strengthening coordination and referral systems between medical and dental providers can help improve oral health outcomes (Crall *et al*., 2005). In a survey of medical providers in North Carolina, about three out of four physicians reported that they were likely to make dental referrals for patients exhibiting early symptoms or at risk of caries (Cruz *et al*., 2004). An important implication of the significant association reported in our study is that the delivery of oral health services alongside PHC may overcome noted disparities in receiving necessary dental care that marginalized populations face. Additionally, the management of dental conditions in the primary care system decreases the likelihood of utilization of emergency medical services for dental ailments, a typically more expensive visit, which favours more efficient resource allocation and a resultant decrease in healthcare costs (Lutfiyya *et al*., 2019; Okunev *et al*., 2022; Wei *et al*., 2022).

A major strength of our study is the generalizability of its findings, supported using data from NHANES, a nationally representative and well-established survey. Additionally, the secondary analysis of pooled data provided sufficient statistical power for our analyses. There are however limitations; for example, since cross-sectional data is largely only suitable for estimating prevalence, most outcomes do not support a high level of causality, only associations. Despite this limitation, the study yields valuable findings for public health leaders responsible for addressing the oral health status of Americans. Given the consistent access to medical providers and their significant influence on overall health, physician-dentist-patient partnerships are crucial for improving the oral health of all Americans. A consideration for future related investigations should involve the incorporation of both individual and area-level data on expanded social determinants of health, dental insurance status, and costs of healthcare in an analysis – this data is not available in NHANES, however. This approach would allow for a more comprehensive evaluation of how these complexity of factors also influence the relationship between access to routine PHC and past-year dental visits.

## Conclusions

We have demonstrated a significant positive association between access to routine PHC and the utilization of dental services among US adults. Specifically, individuals who identified having a regular place to go when they are sick or need health advice were more than twice as likely to have had a dental visit in the past year compared to those without a routine PHC provider. This relationship remained robust even when adjusting for sociodemographic factors and health insurance status. While the study highlights the vital role of PHC as an entry point for dental care, it also reveals persistent disparities. Black non-Hispanic adults were significantly less likely to have had a past-year dental visit than their White counterparts, a finding that held true even among those with health insurance. This underscores that simply having a regular medical provider or insurance coverage is not enough to eliminate racial gaps in oral healthcare utilization. A policy shift where specific service-level integration (such as dental screenings, oral health counselling, and direct referral protocols) are embedded within the routine workflow of primary care clinics could address these disparities and move us beyond the current administrative divide where medical and dental providers often practice in isolation. Such an approach can help reduce dental visit gaps faced by Black US adults, improve access for other underserved populations, and emphasize the importance of preventive dental care.

## Data Availability

The data supporting the findings of this study are included in the published article. Raw data used for the study are publicly available from NHANES and the National Center for Health Statistics (NCHS). Clean datasets generated and/or analysed during the current study are available from corresponding author, upon reasonable request.
